# Supplementary material for: Harmonized nucleoside mass spectrometry enables reproducible cross-platform RNA modification quantification
Source: bioRxiv. 2026 Jul 21:2026.07.17.739095. Preprint. [Version 1] doi: 10.64898/2026.07.17.739095 (PMC13419381; doi:10.64898/2026.07.17.739095)
Supplement: Supplement 1 [file media-1.docx]

**Supporting Information**

Jan Felix Dalwigk^§1^, Kira Kerkhoff^§1^, Oskar Knittelfelder^§2^, Robert L Ross^§3^, Maria Cristina Petrella^4, 5^, Anna Kuśnierczyk^6^, Tulsi Bhandari^7^, Aurore Attina^8^, Alicia Burkard^9^, Carolina Brás-Costa^10^, Michael S. DeMott^11^, Kitty Johnson^12, 13^, Xenia Kerkhoff-Bernaciak^14^, Jennifer Kist^7^, Ken Kögel^15^, Martina Krämer^9^, Ricardo Moreno-Ballesteros^16^, Ganna Podoprygorina^8^, Kaley Simcox^17^, Özge Simsir^15^, Anton Skriba^18^, Sam Wein^19^, Hana Cahova^18^, Benjamin A. Garcia^10^, Mark Helm^9^, Christophe Hirtz^8^, Katharina Höfer^12, 20^, Sebastian Leidel^6, 21^ , Patrick A Limbach^7^, Eva Novoa^4, 22^, Eduard Sabidó^4, 5^, Sabine Schneider ^15^, Philippe Wolff^23^, Peter Dedon^11^, Vivian Cheung^24^ and Stefanie Kaiser*^1^

^§^ these authors contributed equally

* please address your correspondence to [stefanie.kaiser@pharmchem.uni-frankfurt.de](mailto:stefanie.kaiser@pharmchem.uni-frankfurt.de)

^1^ Goethe University Frankfurt, Institute of Pharmaceutical Chemistry, Max-von-Laue-Str. 9, 60438 Frankfurt, Germany

^2^ Bruker Daltonics GmbH & Co. KG, Fahrenheitstr. 4, 28359 Bremen, Germany

^3^ Thermo Fisher Scientific, 10 Maguire Ave., Lexington, MA 01450

^4^ Centre for Genomic Regulation (CRG), The Barcelona Institue of Science and Technology, Dr Aiguader 88, Barcelona 08003, Spain

^5^Universitat Pompeu Fabra (UPF), Barcelona, Spain

^6^ University of Bern, Swiss RNA Mass Spectrometry Platform, Department of Chemistry, Biochemistry and Pharmaceutical Sciences, Freiestrasse 3, 3012 Bern, Switzerland

^7^ Rieveschl Laboratories for Mass Spectrometry, Department of Chemistry, University of Cincinnati, Cincinnati, Ohio 45221, United States

^8^ Plateforme Protéomique Clinique, IRBM – CHU St.-Eloi, Montpellier, France

^9^ Johannes Gutenberg-Universität, Institute of Pharmaceutical and Biomedical Sciences, Staudingerweg 5, 55128 Mainz, Germany

^10^ Department of Biochemistry and Molecular Biophysics, Washington University School of Medicine, St. Louis, Missouri, United States

^11^ Massachusetts Institute of Technology, Department of Biological Engineering, Cambridge, MA 02139, United States

^12^ Phillips University Marburg, Department of Pharmacy, Institute for Pharmaceutical Biology and Biotechnology, 35037 Marburg, Germany

^13^ International Max Planck Research School “Principles of Microbial Life”, Max Planck Institute for Terrestrial Microbiology, Karl-von-Frisch-Straße 10, 35043 Marburg, Germany

^14^ TU Dortmund University, Department of Mathematics, Vogelpothsweg 87, 44227 Dortmund, Germany

^15^ Ludwigs-Maximilians-University Munich, Department of Chemistry, Institute of Chemical Epigenetics, Butenandtstraße 5-13, 81377 Munich Germany

^16^ University of Dundee, School of Life Sciences, Protein Phosphorylation and Ubiquitylation Unit, Dow Street, DD1 5EH Dundee, United Kingdom

^17^ University of Michigan, Department of Chemistry, Ann Arbor, MI 48109, United States

^18^ Czech Academy of Sciences, Institute of Organic Chemistry and Biochemistry, Flemingovo náměstí 2, Prague 6, Czechia

^19^ University of Tuebingen, Department of Computer Science, Maria-von-Linden-Straße 6, 72076 Tübingen

^20^Center for Synthetic Microbiology (SYNMICRO), Phillips University Marburg, Marburg, Germany

^21^University of Bern, Research Group for RNA Biochemistry Department of Biochemistry and Pharmaceutical Sciences, Freiestrasse 3, 3012 Bern, Switzerland (to Sebastian Leidel)

^22^ICREA, Barcelona, 08010, Spain

^23^Université de Strasbourg, CNRS, Architecture et Réactivité de l´ARN, 67084 Strasbourg, France

^24^Brown University. Department of Molecular Biology, Cell Biology and Biochemistry, 70 Ship Street, Providence, RI 02903, United States

**
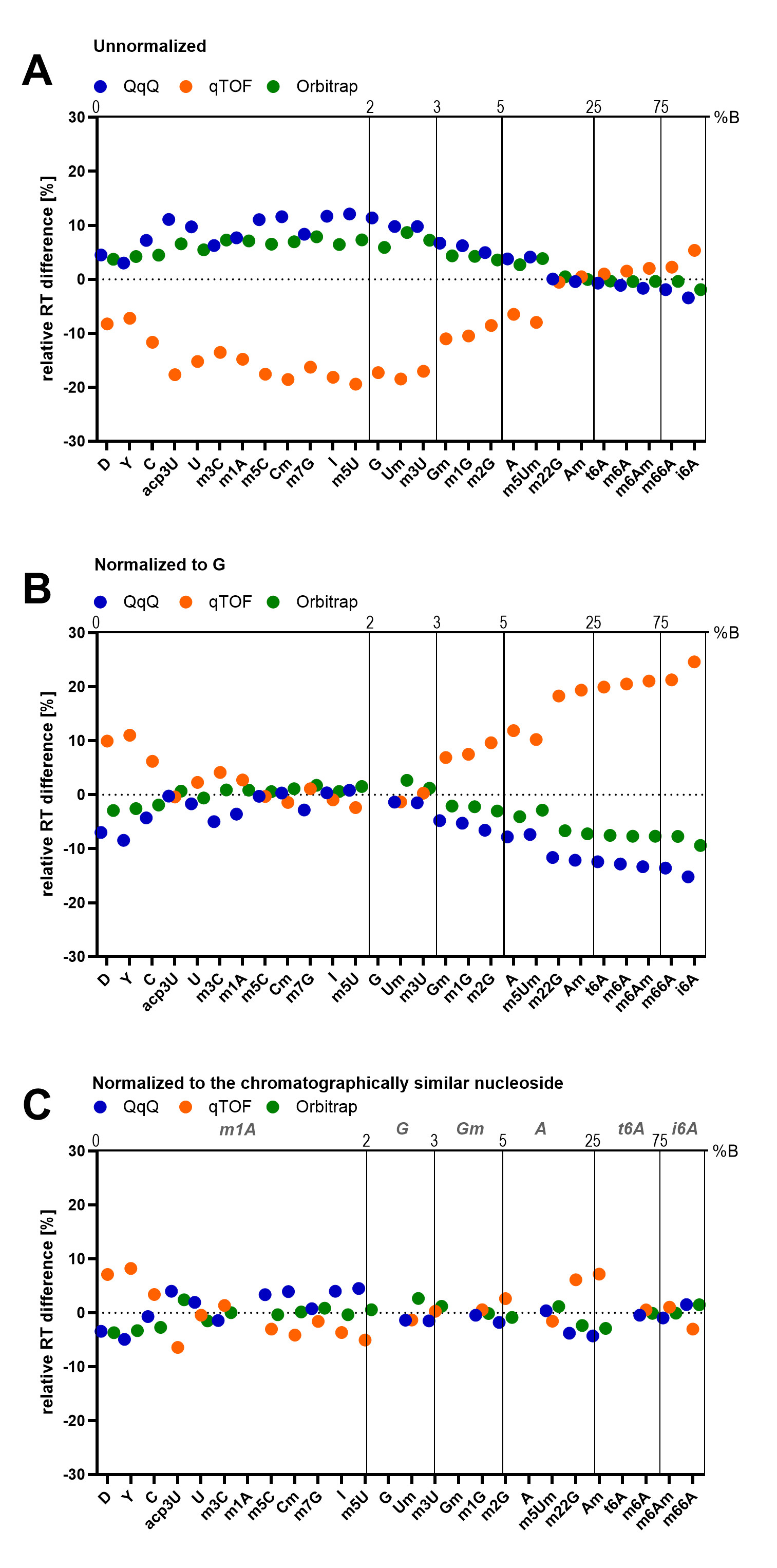
**

**Figure S1** Relative retention time differences calculated from the mean retention time of all systems. The upper y-axis indicates the gradient solvent composition, with the grid indicating changes in solvent composition at the mean retention time of the corresponding modification. **(A)** Relative differences of unnormalized retention times. **(B)** Relative differences of retention times normalized to G. **(C)** Relative differences of retention times normalized to the chromatographically similar nucleoside. The text in cursive indicates which nucleosides were used as a reference for the respective chromatographic section.


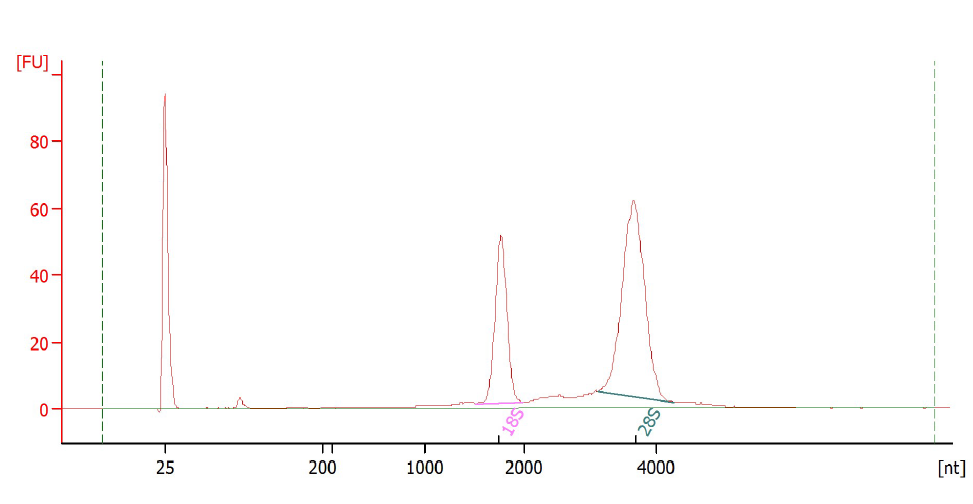


**Figure S2** Electropherogram of small-RNA depleted RNA isolated from Human B-cells (GM12878) analyzed using the Agilent 2100 Bioanalyzer and the RNA 6000 Pico Kit. The prominent peaks correspond to the 18S and 28S ribosomal RNA subunits.
